# Guidelines for Fluorescent Guided Biallelic HDR Targeting Selection With PiggyBac System Removal for Gene Editing

**DOI:** 10.3389/fgene.2019.00190

**Published:** 2019-03-13

**Authors:** Javier Jarazo, Xiaobing Qing, Jens C. Schwamborn

**Affiliations:** Developmental and Cellular Biology, Luxembourg Centre for Systems Biomedicine, University of Luxembourg, Belvaux, Luxembourg

**Keywords:** CRISPR, biallelic, HDR, genome editing, IPSC

## Abstract

The development of new and easy-to-use nucleases, such as CRISPR/Cas9, made tools for gene editing widely accessible to the scientific community. Cas9-based gene editing protocols are robust for creating knock-out models, but the generation of single nucleotide transitions or transversions remains challenging. This is mainly due to the low frequency of homology directed repair, which leads to the screening of a high number of clones to identify positive events. Moreover, lack of simultaneous biallelic modifications, frequently results in second-allele indels. For example, while one allele might undergo homology directed repair, the second can undergo non-homologous end joining repair. Here we present a step-wise protocol for biallelic gene editing. It uses two donors carrying a combination of fluorescent reporters alongside homology arms directed to the same genomic region for biallelic targeting. These homology arms carry the desired composite of modifications to be introduced (homozygous or heterozygous changes). Plus, the backbone of the plasmid carries a third fluorescent reporter for negative selection (to discard random integration events). Fluorescent selection of non-random biallelic targeted clones can be performed by microscopy guided picking or cell sorting (FACS). The positive selection module (PSM), carrying the fluorescence reporter and an antibiotic resistance, is flanked by inverted terminal repeats (ITR) that are recognized by transposase. Upon purification of the clones correctly modified, transfection of the excision-only transposase allows the removal of the PSM resulting in the integration of only the desired modifications.

## Introduction

Disease modeling in vitro had a technological leap with the advent of biotechnology tools such as the induction to pluripotency sates, and the targeted nucleotide modifications by gene editing techniques (Hockemeyer and Jaenisch, 2016). Combining both techniques allows us to validate the effect of disease causing point mutations, as well as the influence of risk variants in the context of a human cell model (Jehuda et al., 2018). Moreover, it can help in the assessment of disease modifiers by introducing mutations that lead to phospho-mimetic or phospho-null protein variants identifying them as novel targets for drug development, without the influence of exogenous or overexpressed sequences (Chen and Cole, 2015).

Even though CRISPR/Cas9 represents the democratization of gene editing tools for most research labs (Jasin and Haber, 2016), certain aspects of the process demonstrated to be cumbersome in practice, such as the number of clones to be screened, reduced biallelic targeting or high on target non-homologous end joining (NHEJ). We previously reported the concept of circumventing these issues by using two constructs targeting the same genomic region but having different positive selection modules (PSM) (Arias-Fuenzalida et al., 2017). These PSM have different fluorescent proteins (namely EGFP and dTomato) allowing the identification of a correct knock-in in both alleles simultaneously. Compared to other systems using only an antibiotic resistance (e.g., puromycin) in the PSM, the use of fluorescent proteins circumvents clones that underwent NHEJ repair in the second allele. The PSM is surrounded by transposon inverted terminal repeats (ITRs) of the piggyBac transposon system for removal of the PSM after selection. The transposase enzyme recognizes these ITRs, excising the sequenced flanked by them reconstituting a TTAA motif in the host genome (Yusa et al., 2011). The use of excision only variants prevents the reintegration of the transposon in the genome (Li et al., 2013).

The previous reported workflow faces challenges when trying to edit genomic regions that present a high density of repetitive elements since it increases the chances of having homologous recombination in other genomic regions (Saito and Adachi, 2016). In our previous work we modeled the influence of the different types of repetitive elements and showed that the presence of repetitive elements of the family Short Interspersed Nuclear Elements (SINE) in the homology arms present higher frequency of random integration (Arias-Fuenzalida et al., 2017). Our model matched the observations previously reported (Ishii et al., 2014). Due to the high content of repetitive elements in mammalian genomes mainly coming from transposable elements integrated during evolution (de Koning et al., 2011), it is in some cases difficult to design homology arms of an appropriate size that are free of repetitive elements. The donor plasmids in the presented design carry negative selection modules for the identification and exclusion of random integration events. As random integration events could occur excluding the negative selection module, we explore adapting the genome engineering pipeline to perform fluorescent-microscopy guided colony picking. The clones selected and picked carry the EGFP+ dTomato+ BFP- fluorescent phenotype. Colonies will have to be PCR screened for detecting the presence of the backbone of the donor construct before continuing with the rest of the workflow. Here we present a detailed protocol for this process.

## Stepwise Protocol

**FIGURE 1:**
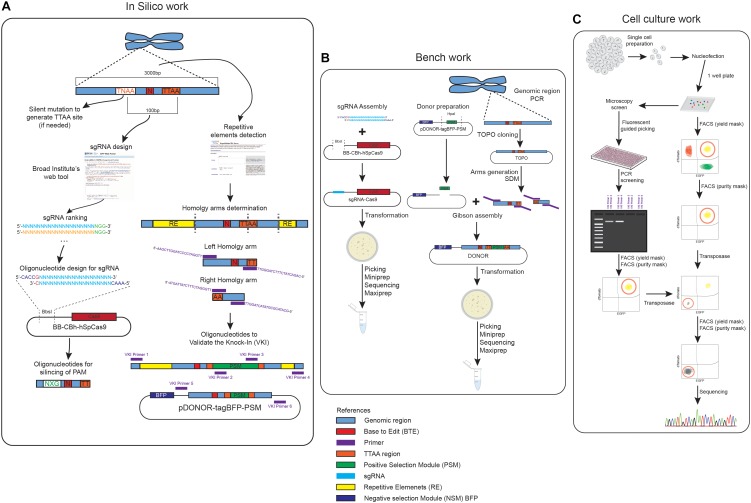
Graphical summary of the pipeline of work. **(A)** Summary of the different steps of in silico work designing the donors, the sgRNA and the oligonucleotides used to generate the constructs. **(B)** Graphical summary of the different steps involved in the generation of the donor and sgRNA construct. **(C)** Different paths of the knock-in and removal of the construct based on a fluorescent guided picking or FACS panclone generation approach.

Please read the entire process before starting since elements listed in the reagents table (Supplementary Table 1) are only those specific for this pipeline (summarized in Figure 1), and common cell culture reagents are not described in detail. Please also use as a reference the Supplementary Table 2 containing the list of primers used in the protocol.

## Concrete Example

In order to easily understand the pipeline of work here presented, we provide an example of a particular Single Nucleotide Polymorphism (SNP) we have edited. The SNP rs45539432^1^ is a transition (c.1366C > T, NM_032409.2) in the PINK1 gene that generates a premature stop codon (p.Gln456Ter, NP_115785.1). This SNP has been linked to early-onset Parkinson’s disease (Hedrich et al., 2006). In this case, the mutations to be corrected are homozygous, hence the design of the homology arms for both donors (carrying EGFP or dTomato in the PSM) is identical. For doing biallelic targeting of heterozygous modifications one of the donors (either the EGFP or dTomato one) should not have the SNP, hence a different homology arm would have to be generated.

## *In Silico* Work

One of the first steps in designing your plasmids for gene editing is the identification of the region of interest to be edited (Figure 1A). It is important to assess if the gene to be modified presents splicing variants that might show unexpected effects of the modification when performing downstream assays for phenotyping. The in silico work is required for designing the donors, the sgRNA and the oligonucleotides used to generate the constructs or to screen the editing process (Figure 1A).

### Designing of the Donors

**FIGURE 2:**
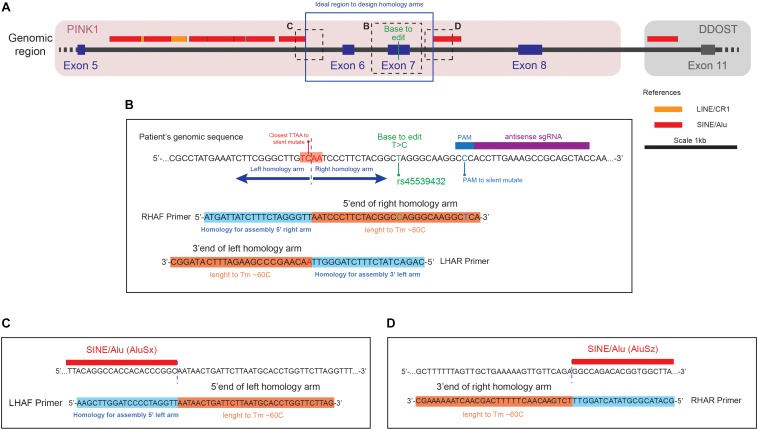
Representation of the genomic region to correct the transition (c.1366T > C, NM_032409.2) in the PINK1 gene **(A)** Genomic region around the PINK1 Q456X mutation identifying the position of the base to edit respect of the repetitive elements in the area. **(B)** Close up of the genomic sequence centered in the Base to Edit (BTE), with the design of the primer Right Homology Arm Forward (RHAF) and Left Homology Arm Reverse (LHAR). Notice that the RHAF primer carries the correction of the BTE and a silent mutation to avoid PAM recognition. Also notice that the LHAR primer carries the silent mutation to generate a TTAA site close to the BTE. **(C)** Close up of the genomic region that is the boundary of the left homology arm, with the design of the Left Homology Arm Forward (LHAF) primer. **(D)** Close up of the genomic region that is the boundary of the right homology arm, with the design of the Right Homology Arm Reverse (RHAR) primer.

For designing the donors, the identification of the Base to Edit (BTE) and the context of the genomic region allows the user to screen for the presence of repetitive elements that could define the boundaries of the homology arms (Figure 2A). Considering a broader genomic region around the desired site for introducing the mutation helps the user to create the entire pipeline for screening the editing process. We recommend the usage of a sequence editor software (SES) such as ApE^2^ or SnapGene^3^ for working with the sequences over all the steps of the design. The steps required for designing the donors can be summarized in: identification of the region to be edited, evaluation of the presence of repetitive elements, identification of a TTAA site and design of primers for generating the arms.

#### Identification of the Region to Be Edited

1. These recommendations and steps are based on the introduction of SNPs in coding regions of the genome. However this technique could also be applied for the introduction of SNPs in non-coding regions.
2. Identify in the genome the position of the BTE (Figure 2A). In case no detailed information is available about the SNP desired to introduce, and only the amino acid change in the protein sequence is known, we recommend following the steps mentioned on Box 1.
  **BOX 1 |** Guidelines for identifying the base to edit (BTE) in the genomic sequence.
  • Use the Nucleotide search tool available in Pubmed to identify the region desired (https://www.ncbi.nlm.nih.gov/nuccore/) Introduce your gene name. Select the RefSeq option and download the NM_file.
  • Open the file in the software and select the coding region (CDS).
  • Then translate your selection and find the amino acid that you want to change and the surrounding sequence of the codon you would like to change.
  • Translate that region and identify the position of the amino acid you want to change with the SNP.
  • We recommend using the SNP website from Pubmed for evaluating the role of the Base to Edit (BTE). If it is an annotated SNP this website would also provide the roles in the different splicing variants of the mRNA.
3. Centered on BTE, select a genomic region that expands 3 kbp upstream and downstream, and transfer this information into a SES.

#### Evaluation of the Presence of Repetitive Elements

1. Upload your sequence in the Repeat Masker tool^4^ for identifying the presence of repetitive elements in the selected region. Repetitive elements should be avoided as much as possible in designing the homology arms.
2. Upload the information of repetitive elements into your region of interest using a SES.
3. Label those regions in your sequence of interest with identifiers of the different types of repetitive elements (Figure 2A).

#### Identification of a TTAA Region in the Vicinity of the BTE

1. Search in the vicinity of the BTE for the presence of a TTAA site, preferably in an exonic region. Ideally the distance between the TTAA site should and the BTE should not be more than 50 bp, making it easier to introduce the SNP with the primers used to amplify the arm in one step. If non TTAA can be found in the vicinity, check if any permutation of TTAA can be achieved by introducing a silent mutation (Figure 2B).
2. Detect the presence of potential silent mutations that can generate the formation of a TTAA site. In order to speed up the process consider using restriction site detection software^5^ and check for restriction enzymes that cut in TTAA site (like MSE I). This point mutation would need to be introduced in the homology arms used in the donor. Control that the introduction of this silent mutation is not drastically changing the codon frequency usage^6^.
3. Select an area of 1 kb upstream and downstream of the TTAA to define the homology arms (Note: In case that the surrounding genomic sequence has presence of repetitive elements reduce the size of the arms to avoid the inclusion of them. Based on the linear optimization calculated in our previous paper a set of guidelines is described in Box 2) (Figure 2B).

**BOX 2 |** Rough guidelines for reducing the size of the homology arms to avoid the repetitive elements (RepEl).

• Do not reduce the size of the homology arm to less than 200 bp even if repetitive elements are present.
• Reduce the size of the arm if repetitive elements of the SINE family are present.
• If there are elements of repeated bases present within the closes 500 bp to the BTE, do not reduce the size of the arms.

### Designing of the sgRNAs

#### Selection of the Guides

1. Upload a sequence of 100 bp upstream and downstream of your BTE to an online tool such as the one provided by the Broad Institute which ranks the sgRNAs based on the on and off targets^7^.
2. Select the best five sgRNA. Selection of the guides should be ideally performed in the exonic area of the gene since silent mutations in the PAM region will need to be introduced. Moreover, the distance between the double strand break (DSB) and the BTE should not be more than 25 bp. In the case a microhomology-mediated end-joining (MMEJ) repair occurs, the integration of the selection cassette can happen without the SNP, if this one is designed outside this limit (Nakade et al., 2014).
3. As previously reported, it is recommended to pick the guides that hit the reading DNA strand of the gene. It is reported to increase efficiency since the RNA polymerase dislodges the bound Cas9 allowing the access to the cell’s repair mechanism (Clarke et al., 2018).

### Oligonucleotides Design

#### Primer Design to Obtain the Arms

1. Use a primer generator tool, such as the one available in the NCBI platform Primer-BLAST^8^.
2. Define sequencing primers (SEQPRA) that can extract 2 kbp upstream and downstream of the BTE. These primers will be used to extract the genomic region to be cloned in a TOPO vector (see section “Preparation of the homology arms template”).
3. Based on the analysis of the presence of repetitive elements, define primers that can ideally extract 1 kb upstream and downstream of the TTAA. In case of repetitive elements, proceed as mentioned in the “Identification of a TTAA region in the vicinity of the BTE” section. The primers to be generated will define the boundaries of your homology arms, namely Left Homology Arm Forward (LHAF), Left Homology Arm Reverse (LHAR), Right Homology Arm Forward (RHAF), and Right Homology Arm Reverse (RHAR) (Figure 2B–D). Each primer possess a homology region to the genomic DNA and a homology to do donor plasmid for assembly. For the homology region in the genomic DNA, consider an amount of bases in the border of your homology arm that generates an oligo with a Tm of 60°C (this can be assessed in the SES) (Figure 2B–D). For the homology region in the donor, overhangs (of 20 bp in length) will need to be added to the designed oligos matching the splitted scaffold after digestion with the restriction enzyme Hpa I (Figure 3A–C). These primers will be used to perform Gibson’s assembly (Gibson, 2011) of the homology arms into the donor scaffold (Figure 3D). The assembly of the homology arms is performed in the TTAA splitting point of the ITR of the donor (Figure 3B).

**FIGURE 3:**
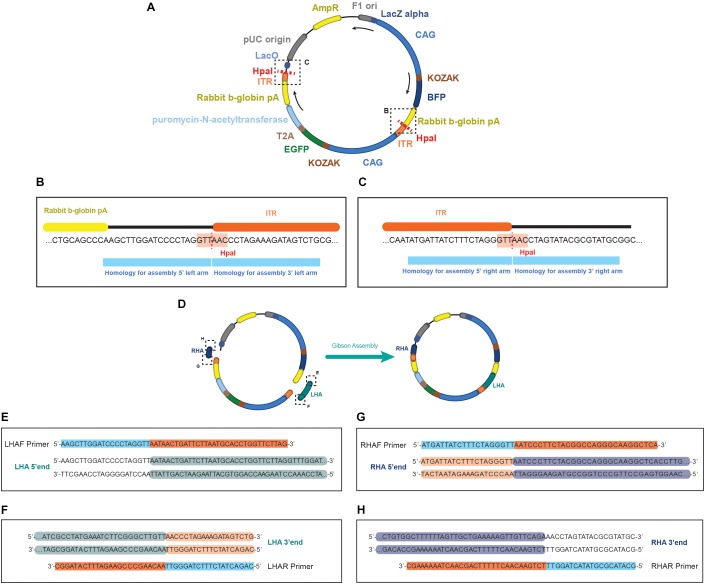
Schematics of the EGFP donor plasmid and the homology in the primers for performing the assembly. **(A)** Representation of the pDONOR-tagBFP-PSM-EGFP with the restriction sites of HpaI. **(B)** Close up of the HpaI region where the left homology arm will be assembled. **(C)** Close up of the HpaI region where the right homology arm will be assembled. **(D)** Representation of the donor after assembly. On each primer from (**E**–**H**) it is represented in light blue the homology to the donor and in orange the homology to the genomic region of the example. Every double stranded DNA section in **E**–**H** represents the sequence of the homology arms to be assembled, showing in green **(E,F)** the genomic sequence incorporated in the left arm, and in dark blue **(G,H)** the genomic sequence incorporated in the right arm. In light orange **(F,G)** the homology in the arms to the ITR sequence of the donor, and unlabeled **(E,H)** the backbone of the donor plasmid.
4. At this point of the primer design consider all the base pair changes that need to be introduced in the donor: BTE, silent mutation of the PAMs, and silent mutation to generate a TTAA (if needed) (Figure 2B). In case that the generation of a silent mutation is needed to produce a TTAA site, we recommend to introduce it with either the LHAR or RHAF primers, depending which is the permutation available: if NTAA or TNAA should be permuted, changes must be made in the LHAR; and if TTNA or TTAN should be changed, the modifications must be in the RHAF. If close to the extremes of the homology arms, consider using these primers for introducing the point mutations (Figure 2B).
5. Four primers will need to be generated (one per extreme of each homology arm) (Figure 2, 3E–H):
  Left Homology Arm Forward (LHAF)
  AAGCTTGGATCCCCTAGGTT (+ sequence into your left homology arm) 3′ end
  Left Homology Arm Reverse (LHAR) at the splitting point of TTAA
  CAGACTATCTTTCTAGGGTT (+ sequence into your left homology arm TTAA site) 3′ end
  Right Homology Arm Forward (RHAF) at the splitting point of TTAA
  ATGATTATCTTTCTAGGGTT (+ sequence into your right homology arm TTAA site) 3′ end
  Right Homology Arm Reverse (RHAR)
  GCATACGCGTATACTAGGTT (+ sequence into your right homology arm) 3′ end

#### Primer Design to Introduce the SNPs

The donor would need to have not only the modification of the BTE that shall be introduced but also the modification of the PAM and a silent mutation to generate a TTAA site (if needed). We recommend to introduce a silent mutation for the PAM of at least 2 different sgRNAs. We recommend to clone the extracted amplicon with the surrounding genomic region to the BTE in a TOPO vector (Zero Blunt^TM^ TOPO^TM^ PCR Cloning Kit, Thermo Fisher Scientific) for doing the steps for inserting the SNPs (see section “Preparation of the homology arms template”). If the SNP are close to the extremes of the arms, they can be introduced with the primers for generating the homology arms (see the previous section). If not, insertion of the SNP could be performed by Site Directed Mutagenesis (SDM, e.g., Q5^®^ Site-Directed Mutagenesis Kit, NEB). Primers would have to be designed to introduce these mutations. Alternatively, this process can be outsourced to a de novo DNA sequence synthesis company (e.g., GeneArt^®^ Gene Synthesis, Thermo Fischer Scientific). In that case, the best option could be to synthesize the left homology arm and the right homology arm independently to then be ligated with the donor scaffold.

#### Oligonucleotide Design for sgRNA

1. We recommend using the plasmid BB-CBh-hSpCas9 (also known as pX330; Addgene #42230) generated by the Zhang lab. The protocol used for cloning the sgRNA into the px330 plasmid has been explained in detailed previously (Ran et al., 2013). Briefly, sgRNA selected from the in silico tool has to be modified in order to be incorporated in the plasmid and its complement has to be also designed (Figure 1A).
2. Selection of only the 20 bp sequence (non-inclusion of the PAM in sgRNA).
  5′-NNNNNNNNNNNNNNNNNNNNNGG-3′
  3′-NNNNNNNNNNNNNNNNNNNNNCC-5′
3. Addition of sticky ends and 5′ G (in case your selected sgRNA starts with a G it is not necessary to add this base).
  5′-CACCGNNNNNNNNNNNNNNNNNNNN-3′
  3′-CNNNNNNNNNNNNNNNNNNNNCAAA-5′

#### Oligonucleotide Design for Validating the Knock-In (VKI)

In order to validate the knock-in, a set of primers has to be designed to verify the right and left junctions between the PSM and the genomic DNA. The left junction forward primer (VKI Primer 1) and the right junction reverse primer (VKI Primer 4) depend on the genomic region of interest (Figure 1A). We recommend designing oligonucleotides in the genomic region at a distance of 500 bp from the junction between the homology arms and genomic DNA for VKI Primer 1 and 4. Alternatively, the same SEQPRA designed in section “Primer design to obtain arms” could be used. For the left junction reverse primer (VKI Primer 2) and the right junction forward primer (VKI Primer 3) we recommend using:

VKI Primer 2
5′-AGATGTCCTAAATGCACAGCG-3′
VKI Primer 3
5′-CGTCAATTTTACGCATGATTATCTTTAAC-3′

Plus, a set primers to obtain an amplicon expanding from the PSM to the backbone of the plasmid to identify random integration events that might have left out the BFP during the integration process. For detecting the presence of the backbone of the plasmid, we recommend designing the left backbone forward primer (VKI Primer 5) and the right backbone reverse primer (VKI Primer 6) (Figure 1A):

VKI Primer 5
5′-GCTGCCTATCAGAAGGTGGTG-3′
VKI Primer 6
5′-GCAGCCACTGGTAACAGGAT-3′

#### Oligonucleotide Design for Final Sequencing

An oligonucleotide (SEQPR) at a distance of around 100 bp from the BTE has to be designed for doing the final sequencing of the edited clone.

## Bench Work

The steps performed in this section are summarized in Figure 1B

### Generation of the Guides

This protocol established by the Zhang lab has been explained in detail previously (Ran et al., 2013). Here we summarize the steps needed for the generation of the sgRNAs.

#### Preparation of px330 Scaffold

1. Digest the vector px330 with Bpil (FastDigest, Thermo Fisher Scientific) for 3 h at 37°C for complete digestion. Prepare a maximum of 1 μg of DNA per single reaction:

**Table:** 

Solution	Volume (μl)
px330	Maximum 1 μg per reaction
Bpil FD	1
Fast Digest buffer (10x)	2.5
H2O	q.s.

Total	25
2. Column purify the digestion product (e.g., with QIAquick PCR Purification Kit, Qiagen) assume a 50% lost in column purification and elute in a volume of nuclease free water.
3. Determine purified plasmid concentration (e.g., by NanoDrop Spectrophotometer).

#### Annealing of sgRNA Oligonucleotides

1. Prepare annealing buffer (AB) using the T4 DNA ligase kit (NEB) at a ratio 1:7 (T4 ligation buffer: H2O). The mix can be stored at −20°C.
2. Example 100 μL of T4 ligation buffer +700 μL of nuclease free water.
3. Prepare annealing reaction.

**Table:** 

sgRNA oligo forward (100 μM)	10 μL
sgRNA oligo reverse (100 μM)	10 μL
Annealing buffer	80 μL

Total	100 μL
4. Annealing. Use a ramp protocol for annealing in thermocycler.

**Table:** 

	Temperature (C)	time	delta T (C)
1	95	5 minutes	na
2 (loop 14x)	95	1 min	−5°C
3	25	HOLD	Na

#### Ligation of Annealed Oligonucleotides and px330

1. Set the ligation reaction as bellow.

**Table:** 

Solution	Volume (μl)
Digested px330	Total 112 ng
annealed oligos	2
T4 ligase	1
T4 ligase buffer	2
H2O	q.s.

Total	20
2. Incubate at RT for 1 h.
3. Transform One Shot^TM^ TOP10 Chemically Competent E. coli (Thermo Fisher Scientific) with 10 μL of ligation following manufacturer’s protocol.
4. Grow in 1 LB agar plate (supplemented with ampicillin) per sample using 450 μL of the culture tube.

#### Picking of Colonies and Sequencing

1. From the transformed plate pick 5 colonies per sample and grow in a volume of 2 ml LB supplemented with ampicillin overnight in shaking conditions. The plates can be stores at 4°C awaiting screening results.
2. The following day from the same tube per sample make a glycerol stock using 500 μL of culture plus 500 μL of 50% glycerol, and purified the plasmid with a miniprep kit (e.g., QIAprep Spin Miniprep Kit, Qiagen) using 1 mL of culture according to manufacturer instructions but eluting in 50 μL of nuclease free water.
3. Send 20 μL of the miniprep sample to sequence with the following primer:
  5′-GAGGGCCTATTTCCCATGATTCC-3′

### Generation of the Donor

#### Preparation of the Donor Scaffold DNA

1. Prepare the donor scaffold DNA with a maxiprep (e.g., HiSpeed Plasmid Maxi Kit, Qiagen).
2. Determine plasmid concentration (e.g., by NanoDrop Spectrophotometer).
3. Digest the DNA with HpaI enzyme (NEB). Bulk preparations are recommended.

**Table:** 

Reagent	Volume for 1 reaction
DNA	Volume of 1 μg (per each reaction)
HpaI (5k unit/ml)	1 μl
NEB buffer 4 (10×)	2 μl
H20	Bring up to 20 μl

Total	20 μl
4. Incubate for 2 h at 37°C in incubator.
5. Purify the digestion product using a column purification kit (e.g., with QIAquick PCR Purification Kit, Qiagen).

#### Preparation of the Homology Arms Template

If the homology arm generation was outsourced to a de novo DNA sequence synthesis company skip this section.

1. Define the parental line you want to edit and purify genomic DNA. Karyotype the line before you start with the editing process, and if possible sequence specific regions that might be interesting for your specific disease modeling to discard that the parental has unexpected alterations from the beginning.
2. Using as template the parental line you want to edit, amplify the genomic DNA with the SEQPRA primers designed in step 2 of “Primer design to obtain the arms.” For this step use a high fidelity polymerase (e.g., PrimeSTAR^®^ GXL DNA Polymerase, Takara).

**Table:** 

Reagent	Volume for 1 reaction
Genomic DNA	Volume of 100–200 ng
GXL polymerase (10U/μl)	1 μl
GXL polymerase buffer (5x)	10 μl
dNTPs (2.5mM)	4 μl
Primer forward (10 μM)	2 μl
Primer reverse (10 μM)	2 μl
H20	to 50 μl

Total	50 μl
3. Define the amplification PCR protocol depending on enzyme extension temperature, enzyme synthesis speed and template length. Normally 30 cycles of amplification is enough.
4. Verify the amplified product by agarose gel electrophoresis. Use only 5 μl.
5. Purify the rest of the PCR product using a column purification kit (e.g., with QIAquick PCR Purification Kit, Qiagen).
6. Clone the PCR product of one reaction into a TOPO vector (Zero Blunt^TM^ TOPO^TM^ PCR Cloning Kit, Thermo Fisher Scientific) following manufacturer’s instructions. For choosing the right TOPO consider the terminal ending activity of the polymerase. Most high fidelity polymerases generate blunt end products.
7. After cloning, the TOPO-gDNA vector should be restriction mapped and sequenced. This allows to define if there are SNP variants in your region of interest. Special consideration should be placed in Cas9 binding sites and TTAA region.

#### Introduction of Mutations

As explained previously in the section “Primer design to introduce the SNPs,” SNPs to be introduced in the region of interest should be performed on the TOPO vector generated in the previous section using SDM or introduced with the primers designed to obtain the arms (see step 4 of “Primer design to obtain the arms”).

#### Preparation of the Homology Arms for Assembly

The assembly of the donor DNA is performed using Gibson assembly (Gibson, 2011). Homology between the homology arms and the scaffold is required. Use the primers designed in step 5 of section “Primer design to obtain the arms” to incorporate the arms in the HpaI splitting sites of the scaffold donor.

1. Amplify, verify, and purify the homology arms from the mutated TOPO or the synthetic DNA from a company following steps 2–5 from the previous section but using the pair of primers LHAF and LHAR (to obtain the left arm), and the pair of primers RHAF and RHAR(to obtain the right arm) in two separate PCR reactions.
2. Assemble the left homology arm, the right homology arm and the scaffold DNA prepared in step 5 of “Preparation of the donor scaffold DNA” section using Gibson Assembly^®^ Master Mix (NEB) following manufacturer’s protocol. We recommend the usage of online calculators for stablishing the stoichiometry of the ligation pieces^9^ or designing the ligation with assembly tools^10^. Having the right proportion of the different elements during the assembly drastically increases the efficiency.
3. Transform NEB^®^ 10-beta Competent E. coli (NEB) with 2 μL of the assembly following manufacturer’s protocol.
4. Grow in 1 LB agar plate (supplemented with ampicillin) per sample using 450 μL of the culture tube.
5. Follow the steps 1 and 2 in section “Picking of colonies and sequencing” for picking and plasmid glycerol stock. In this case pick 10 colonies per donor.
6. Screen by restriction mapping and sequence those that present an expected map.
7. Perform a maxiprep (e.g., HiSpeed Plasmid Maxi Kit, Qiagen) of sequence confirmed donors.
8. Measure plasmid concentration and take it to a 500 ng/ul working concentration.

### Preparation of Transposase mRNA

Removal of the selection cassette after can be performed by the transfection of mRNA. This mRNA can be either in vitro generated in the lab using the template described in Yusa et al., 2011; Yusa, 2013 (construct pCMV-HAhyPBase); or Li et al., 2013; or it can be commercially acquired^11^.

1. PCR amplify the coding sequence of codon optimized hyperactive transposase piggybac from Trichoplusia ni (Yusa et al., 2011) or the excision-only mutant (Li et al., 2013) to incorporate the T7 promoter:
  T7-transposase_F
  5′-GAAATTAATACGACTCACTATAGGGCCGCCACCATGGGCAGCAGCCTGGAC-3′
  T7-transposase_R
  5′-GGCAAACAACAGATGGCTGG-3′
2. Column purify PCR product.
3. Use the PCR product as a template for in vitro transcription with a mMESSAGE mMACHINE^TM^ T7 Transcription Kit (Thermo Fisher Scientific) following manufacturer’s protocol.
4. Polyadenylate the transcript with a Poly(A) tailing kit (Thermo Fisher Scientific) and purified with a MEGAclear^TM^ Transcription Clean-Up Kit (Thermo Fisher Scientific).

### *In vitro* Testing of sgRNA Efficiency

*In silico* efficiency of the guides can be further tested with in vitro assays for EGFP reconstitution as described in Mashiko et al. (2013); Mashiko et al. (2014).

## Cell Culture Work

Edition of cells under this protocol is normally performed in hiPSCs cultured in Matrigel (Corning) coated plates with daily changes of Essential 8 media (Thermo Fisher Scientific) supplemented with 1% Penicillin/Streptomycin. Cells are normally passaged and handled as single cells (Figure 1C). This is performed by using Accutase (Thermo Fisher Scientific) and supplementing the Essential 8 for 24 h after passaging with ROCK inhibitor to prevent apoptosis (Y-27632, Merck Millipore).

### Nucleofection of Parental Cells and Selection

Nucleofection is performed using a 4D-Nucleofector^TM^ X Unit (Lonza) and the P3 Primary Cell 4D-Nucleofector^TM^ X Kit L (Lonza). Expansion of the cells pre-nucleofection can be performed in flasks or 10 cm dishes. Seeding of the cells after nucleofection should be done in a Matrigel pre-coated 1 well plate Nunc^TM^ OmniTray^TM^ (Thermo Fisher Scientific) to allow the possibility of doing fluorescence guided picking. Ideally, do 2 different batches of electroporation each with a different sgRNA-Cas, and make five electroporations per sgRNA-Cas.

1. Expand parental cells to have a million cells per electroporation. Cells have to be freshly plated (no more than 4 days after the previous passage).
2. Detach cells using Accutase, neutralize using DMEM/F12 (Thermo Fisher Scientific), centrifuge (200 × *g* 3 min), resuspend in 5 ml of media, and count.
3. Transfer 1 million cells into a 1.5 ml Eppendorf tube and centrifuge (200 ×*g*, 3 min). Resuspend with 100 μl of supplemented nucleofector solution (see manufacturer’s protocol) and transfer to electroporation cuvette.
4. Electroporate using the “human stem cells h9” protocol that performs the CB150 pulse in the 4D-nucleofector (Lonza). Use 1.5 μg of each donor and 2.5 μg of sgRNA-Cas per electroporation.
5. After pulse, add 1 ml of Essential 8 media supplemented with ROCK inhibitor (10μM) to the nucleofector cuvette and then transfer the content of one cuvette to one Matrigel pre-coated 1 well plate.
6. Culture on media supplemented with ROCK inhibitor for 24 h.
7. Culture until small to medium size colonies have grouped and supplement media with Puromycin (0.25 μg/ml). This concentration can be adjusted in the next 3 days depending on the survival increasing it to 0.5 μg/ml.
8. Two days after antibiotic treatment small colonies should emerge and continue to populate the plate. Selection with Puromycin should be maintained through the next stages of culturing until treatment with transposase.

### FACS or Fluorescence Guided Picking

**FIGURE 4:**
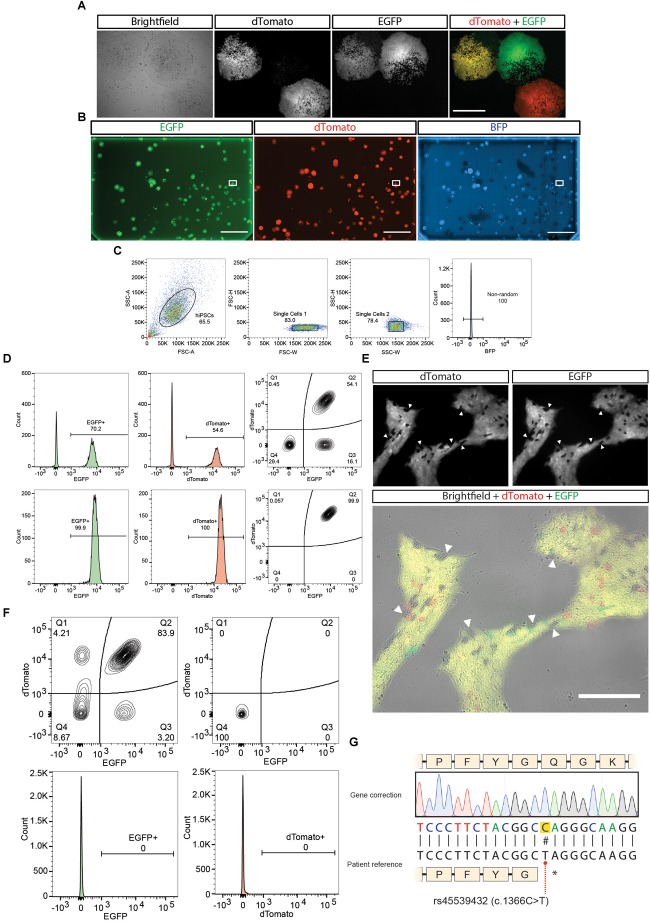
Cell culture work of the gene correction example. **(A)** Representative images of hiPSCs colonies expressing the different possible outcomes of gene modification. Scale bar = 500 μm. **(B)** A one-well plate screened for detecting correct biallelic targeting (dTomato+/EGFP+/BFP-). Bounding box shows selected isolated region. Scale bar = 2 cm. **(C)** Single cell isolation gating strategy performed, plus exclusion of BFP+ cells from an expanded culture derived from the selected region in **(B)**. **(D)** First (upper panel) and second (lower panel) purity sort for purifying dTomato+/EGFP+/BFP- cells. **(E)** Removal of the positive selection module after treatment with transposase. **(F)** First sort after transposase induction (top left panel). Last purity sort before sequencing (*n* = 3) (top right panel and bottom panels). **(G)** Sequencing results of the gene correction of the patient line.

As explained in the introduction, the presence of repetitive elements in the homology arms increases the chances of having random integration events. Even though the usage of a BFP in the backbone of the plasmid is used to detect these events, there is still a chance of having integration in an unspecific site that does not include the BFP. For this reason we recommend if the presence of repetitive elements in the homology arm cannot be avoided during the design phase to perform fluorescence guided picking of EGFP+/Dtomato+/BFP- colonies rather than generating a panclone through FACS (Figure 1C). It is important to notice that one of the advantages of using fluorescence reporters with strong promotors as in these donors is that some cells not harboring the PSM (and hence not having resistance to Puromycin) can still survive the Puromycin treatment if in the context of a colony containing resistant cells. These WT cells could be easily carried over, reducing the specificity of a drug based approaches (Figure 4A).

#### Fluorescence Guided Picking

1. Colonies having a size of around 0.25 mm are suitable for fluorescence guided picking. This time will depend on the dividing rate of the iPSCs you are working with.
2. Approximately one week after selection screen the plate for fluorescent colonies. Colonies having an EGFP+/Dtomato+/BFP- fluorescence should be picked and plated in on well of a Matrigel pre-coated 96 well plate (Figure 4B). Picking should be performed using a pipette tip in an angle to gently detach the colony from the plate. It is recommended to have Essential 8 media supplemented with ROCK inhibitor (10μM) in the source and receiver plate. The media with ROCK inhibitor should be added to the source plate one hour prior picking. It is recommended to pick all the colonies present in the plate (on average 1–5 colonies/plate are expected). We recommend picking all colonies resenting the EGFP+/Dtomato+/BFP- pattern. We discourage picking colonies with mixed fluorescent patterns, where some cells might be EGFP+ or Dtomato+ since the validation by PCR (see step 5) might give conflicting results. It is fine to pick EGFP+/Dtomato+/BFP- that present some WT cells since they can be remove in the first sort (see next section).
3. Depending on the initial size of the colony picked, an average waiting time of 3 days is enough to obtain sufficient material for expanding this one well of a 96 well into 4 wells. Two of these wells are going to be used for genomic DNA extraction.
4. Genomic DNA extraction is done using QuickExtract^TM^ DNA Extraction Solution (Epicenter) and following manufacturer’s indications.
5. Perform a PCR for detecting events of random integration without BFP using the plasmid designed in section “Oligonucleotide design for validating knock-in.” Both pairs of primers should be use for detecting random integration by assessing the presence of elements of the donor backbone on either side of the homology arms. For assessing this use primers pair VKI Primer 5 and VKI Primer 2, and primers pair VKI Primer 3 and VKI Primer 6. At this point it would be also appropriate to control the junctions between the homology arms and the genomic region. For assessing this, use the primers pair VKI Primer 1 and VKI Primer 2 to detect the left junction; and primers pair VKI Primer 3 and VKI Primer 4 to detect the right junction.
6. Clones presenting backbone of the plasmid integrated in the genome or lacking one of the junctions should be discarded.
7. Expansion of the clones having the correct profile needs to be done before discarding the presence of unedited cells by FACS.
8. For details of the sorting procedure please see the next section

#### FACS

The starting point for the cell sorting can be from the puromycin selected culture after one/two weeks of nucleofection or from the isolated clone obtained from fluorescence guided picking. In case of the former, it is expected to see around 2% of cells presenting an EGFP+/Dtomato+/BFP- pattern (Arias-Fuenzalida et al., 2017). In the case of the latter, positive cells can range between 50 and 100% depending on the number of WT cells that could be forming the colony (Figure 4C,D). If using a BD FASC Aria II for performing the cell sorting, we recommend using an 85 μm nozzle, the 2.0 neutral density filter and the refrigeration system set at 4°C.

1. Treat for 5 min the iPSCs culture with Accutase to generate a single cell suspension. Some iPSCs lines might need different times for detachment. In that case it is better to increase the time of incubation with Accutase rather than exerting mechanical force by pipetting.
2. Accutase activity should be stop and the sample centrifuged as explained in section “Nucleofection of parental cells and selection”
3. Cells are resuspend in sorting solution (2% bovine serum albumin, 1% Penicillin/streptomycin in PBS) supplemented with ROCK inhibitor (30 μM). Dilution of the cells can be afterwards adjusted based on the rate of events observed.
4. Filter the cell suspension using a 20 μm pre-separation filter (Miltenyi) to remove doublets.
5. Cells should be sorted into sorting solution supplemented with ROCK inhibitor (30 μM). We recommend performing a first round of sorting using a “yield” mask to maximize the recovery of EGFP+/Dtomato+/BFP- fluorescent cells. In successive runs, change instead to a “4-way purity mask” (Figure 4D).
6. To determine the last run of sorting pre-transposase transfection we recommend taking a sample of the cell suspension and staining it with a blue dead cell dye (e.g., SYTOX^TM^ Blue Dead Cell Stain, Thermo Fisher Scientific) to discard the presence of large debris that might be interpreted as non-edited cells still present in the culture (Figure 4D).
7. Only after the culture presents ∼100% of EGFP+/Dtomato+/BFP- fluorescent cells it is possible to continue to the transposase transfection. After having a dead cell stained sample which presents ∼100% of EGFP+/Dtomato+/BFP- you can continue to the next section.

### Removal of Positive Selection Module With Transposase

1. The selected polyclone or clone can be plated on two wells of a 6 well plate at a density of have a million cells per well one day before transfection.
2. Transfect the transposase mRNA generated in section “Preparation of transposase mRNA” with Stemfect^TM^ RNA transfection kit (Stemgent) according to manufacturer’s protocol.
3. The next day repeat the transfection.
4. Allow the culture to recover and proliferate. Once the culture is ready to passage, expand until having 2 10 cm dishes at 80% confluency for sorting (Figure 4E). Notice that either the EGFP or the dTomato PSM can be removed first.

### FACS of Cells That Underwent PSM Removal

Follow the same instructions performed in section “FACS” to obtain a single cell suspension ready for sorting. We recommend performing a first round of sorting using a “yield” mask to maximize the recovery of EGFP-/Dtomato-/BFP- fluorescent cells. An efficiency between 5 and 15% should be expected of the removal of cells (Figure 4F). In successive runs, change instead to a “4-way purity mask.” Only after the culture presents 100% of EGFP-/Dtomato-/BFP- fluorescent cells, a final sequencing should be performed (Figure 4F).

### Confirming by DNA Sequencing

1. Use the primers designed for extracting the amplicon region to clone in the TOPO vector named SEQPRA to generate an amplicon expanding from genomic regions outside the homology arms.
2. Sequence the amplicon with the primer SEQPR designed in the “Oligonucleotide design for final sequencing” section and confirm the presence of the change in the BTE (Figure 4G) as well as the silent mutations for the PAMs and TTAA generation.

## Conclusion

Gene editing technologies, specifically CRISPR/Cas9 gene editing, are starting to revolutionize biological sciences to a similar extent as the invention of PCR or hiPSCs did (Ledford, 2015). The real and potential applications of gene editing range from increasing crops and livestock yields to disease diagnostics and gene drives (Doudna and Barrangou, 2016). In the context of medicine, one of the applications of this technique is disease modeling by targeted modifications of the genome. Here we covered a procedure for the generation of isogenic lines for doing disease modeling, which allows to evaluate the influence of a specific point mutation or the effect of the genetic background of the patient in the onset and progression of a disease (Bolognin et al., 2018). Due to the high number of clones that needed to be screened to obtain a positive one (Paquet et al., 2016), alternatives techniques were needed by the research community.

We provided a detailed guideline for our previous work (Arias-Fuenzalida et al., 2017), adding the alternative path of doing fluorescence guided picking when the design of the homology arms cannot avoid the inclusion of repetitive elements. We consider that the strength of our protocol resides in the certainty obtained after reaching the different milestones of the pipeline, and the simultaneous biallelic targeting to generate isogenic lines for disease modeling.

## Author Contributions

JJ, XQ, and JS contributed conception and design of the protocol. JJ wrote the first draft of the manuscript. All authors contributed to manuscript revision, and read and approved the submitted version.

## Conflict of Interest Statement

The authors are inventors in patent PCT/EP2017/051889.

## Footnotes

1 https://www.ncbi.nlm.nih.gov/projects/SNP/snp_ref.cgi?rs=45539432

2 http://jorgensen.biology.utah.edu/wayned/ape/

3 http://www.snapgene.com/

4 http://repeatmasker.org/

5 http://resitefinder.appspot.com/

6 https://www.genscript.com/tools/codon-frequency-table

7 https://portals.broadinstitute.org/gpp/public/analysis-tools/ssgRNA-design

8 https://www.ncbi.nlm.nih.gov/tools/primer-blast/

9 http://nebiocalculator.neb.com/#!/ligation

10 http://nebuilder.neb.com/

11 https://www.transposagenbio.com

## References

[B1] Arias-Fuenzalida J. Jarazo J. Qing X. Walter J. Gomez-Giro G. Nickels S. (2017). FACS-assisted CRISPR-Cas9 genome editing facilitates parkinson’s disease modeling. *Stem Cell Rep.* 9 1423–1431. 10.1016/j.stemcr.2017.08.026 28988985 PMC5830965

[B2] Bolognin S. Fossépré M. Qing X. Jarazo J. Ščančar J. Moreno E. L. (2018). 3D cultures of parkinson’s disease-specific dopaminergic neurons for high content phenotyping and drug testing. *Adv. Sci.* 6:1800927. 10.1002/advs.201800927 30643711 PMC6325628

[B3] Chen Z. Cole P. A. (2015). Synthetic approaches to protein phosphorylation. *Curr. Opin. Chem. Biol.* 28 115–122. 10.1016/j.cbpa.2015.07.001 26196731 PMC4624483

[B4] Clarke R. Heler R. Macdougall M. S. Yeo N. Chavez A. Regan M. (2018). Enhanced bacterial immunity and mammalian genome editing via RNA-polymerase-mediated dislodging of Cas9 from double-strand DNA breaks. *Mol. Cell* 71 42.e8–55.e8. 10.1016/j.molcel.2018.06.005 29979968 PMC6063522

[B5] de Koning A. P. Gu W. Castoe T. A. Batzer M. A. Pollock D. D. (2011). Repetitive elements may comprise over two-thirds of the human genome. *PLoS Genetics* 7:e1002384. 10.1371/journal.pgen.1002384 22144907 PMC3228813

[B6] Doudna J. A. Barrangou R. (2016). Applications of CRISPR technologies in research and beyond. *Nat. Biotechnol.* 34:933. 10.1038/nbt.3659 27606440

[B7] Gibson D. G. (2011). Methods in enzymology. *Methods Enzymol.* 498 349–361. 10.1016/B978-0-12-385120-8.00015-2 21601685 PMC7149801

[B8] Hedrich K. Hagenah J. Djarmati A. Hiller A. Lohnau T. Lasek K. (2006). Clinical spectrum of homozygous and heterozygous PINK1 mutations in a large german family with parkinson disease: role of a single hit? *Arch. Neurol.* 63 833–838. 10.1001/archneur.63.6.833 16769864

[B9] Hockemeyer D. Jaenisch R. (2016). Induced pluripotent stem cells meet genome editing. *Cell Stem Cell* 18 573–586. 10.1016/j.stem.2016.04.013 27152442 PMC4871596

[B10] Ishii A. Kurosawa A. Saito S. Adachi N. (2014). Analysis of the role of homology arms in gene-targeting vectors in human cells. *PLoS One* 9:e108236. 10.1371/journal.pone.0108236 25250686 PMC4176728

[B11] Jasin M. Haber J. E. (2016). The democratization of gene editing: insights from site-specific cleavage and double-strand break repair. *DNA Repair* 44 6–16. 10.1016/j.dnarep.2016.05.001 27261202 PMC5529214

[B12] Jehuda R. B. Shemer Y. Binah O. (2018). Genome editing in induced pluripotent stem cells using CRISPR/Cas9. *Stem Cell Rev. Rep.* 14 323–336. 10.1007/s12015-018-9811-3 29623532

[B13] Ledford H. (2015). CRISPR, the disruptor. *Nat. News* 522:20. 10.1038/522020a 26040877

[B14] Li X. Burnight E. R. Cooney A. L. Malani N. Brady T. Sander J. D. (2013). piggyBac transposase tools for genome engineering. *Proc. Natl. Acad. Sci.* 110 E2279–E2287. 10.1073/pnas.1305987110 23723351 PMC3690869

[B15] Mashiko D. Fujihara Y. Satouh Y. Miyata H. Isotani A. Ikawa M. (2013). Generation of mutant mice by pronuclear injection of circular plasmid expressing Cas9 and single guided RNA. *Sci. Rep.* 3:3355. 10.1038/srep03355 24284873 PMC3842082

[B16] Mashiko D. Young S. A. Muto M. Kato H. Nozawa K. Ogawa M. (2014). Feasibility for a large scale mouse mutagenesis by injecting CRISPR/Cas plasmid into zygotes. *Dev. Growth Diff.* 56 122–129. 10.1111/dgd.12113 24372541

[B17] Nakade S. Tsubota T. Sakane Y. Kume S. Sakamoto N. Obara M. (2014). Microhomology-mediated end-joining-dependent integration of donor DNA in cells and animals using TALENs and CRISPR/Cas9. *Nat. Commun.* 5:5560. 10.1038/ncomms6560 25410609 PMC4263139

[B18] Paquet D. Kwart D. Chen A. Sproul A. Jacob S. Teo S. (2016). Efficient introduction of specific homozygous and heterozygous mutations using CRISPR/Cas9. *Nature* 533 125–129. 10.1038/nature17664 27120160

[B19] Ran A. F. Hsu P. D. Wright J. Agarwala V. Scott D. A. Zhang F. (2013). Genome engineering using the CRISPR-Cas9 system. *Nat. Protoc.* 8 2281–2308. 10.1038/nprot.2013.143 24157548 PMC3969860

[B20] Saito S. Adachi N. (2016). Advances in the development of gene-targeting vectors to increase the efficiency of genetic modification. *Biol. Pharm. Bull.* 39 25–32. 10.1248/bpb.b15-00701 26725425

[B21] Yusa K. (2013). Seamless genome editing in human pluripotent stem cells using custom endonuclease–based gene targeting and the piggyBac transposon. *Nat. Protoc.* 8 2061–2078. 10.1038/nprot.2013.126 24071911

[B22] Yusa K. Zhou L. Li M. Bradley A. Craig N. L. (2011). A hyperactive piggyBac transposase for mammalian applications. *Proc. Natl. Acad. Sci.* 108 1531–1536. 10.1073/pnas.1008322108 21205896 PMC3029773

